# Clinical features and development of Sepsis in *Klebsiella pneumoniae* infected liver abscess patients: a retrospective analysis of 135 cases

**DOI:** 10.1186/s12879-021-06325-y

**Published:** 2021-06-23

**Authors:** Shixiao Li, Sufei Yu, Minfei Peng, Jiajia Qin, Chunyan Xu, Jiao Qian, Minmin He, Peng Zhou

**Affiliations:** 1 Department of Clinical Microbiology Laboratory, Taizhou Hospital of Zhejiang Province affiliated to Wenzhou Medical University, Taizhou, Zhejiang province China; 2 Department of Pharmacy, Taizhou Hospital of Zhejiang Province affiliated to Wenzhou Medical University, No. 150, Ximen Road of Linhai, Taizhou, 317000 Zhejiang Province China

**Keywords:** Pyogenic liver abscess, *Klebsiella pneumoniae*, Sepsis, Metastatic infection

## Abstract

**Background:**

*Klebsiella pneumoniae* is a primary pathogen of pyogenic liver abscess (PLA). However, little data are available on combination with sepsis. In this study, we aimed to evaluate the clinical characteristics and prognostic differences of PLA patients with sepsis.

**Methods:**

This retrospective cohort study was conducted to investigate 135 patients with confirmed *Klebsiella pneumoniae*-caused liver abscesses (KPLA) from a tertiary teaching hospital, from 2013 to 2019. The patients were divided into two groups, KPLA with sepsis and KPLA without sepsis. The demographic characteristics, clinical features as well as laboratory and microbiologic findings were analyzed.

**Results:**

A total of 135 patients with KPLA were analyzed. The mean age of patients was 60.9 ± 12.7 years, and the percentage of men was 59.3%. Among them, 37/135 (27.4%) of patients had sepsis and the mortality rate was 1.5%. The most common symptom was fever (91.1%). KPLA patients with sepsis had a significantly higher proportion of frailty, diarrhea, fatty liver, chronic renal insufficiency, and hepatic dysfunction compared to KPLA patients without sepsis (*p* < 0.05). Antibiotic therapy and percutaneous drainage were most frequently therapeutic strategy. Furthermore, the incidences of sepsis shock and acute respiratory distress syndrome were higher in the sepsis group compared to the non-sepsis group. As for metastatic infections, the lung was the most common site. In addition, KPLA patients with sepsis showed respiratory symptoms in 11 patients, endophthalmitis in 4 patients, and meningitis in 1 patient.

**Conclusion:**

Our findings emphasize that KPLA patients combined with or without sepsis have different clinical features, but KPLA patients with sepsis have higher rates of complications and metastatic infections. Taken together, further surveillance and control of septic spread is essential for KPLA patients.

## Introduction

Pyogenic liver abscess (PLA) is a common intraabdominal infectious disease, which is caused by various bacteria. Recent findings showed a much higher incidence of PLA in Taiwan (17.6 per 100,000 individuals) than that in northeast China (5.7 per 100,000 individuals), and the United States (3.59 per 100,000 individuals) [1]. With the recent advances in diagnostic and therapeutic capability, the fatality rate has gradually declined (7.8 to 28.6%) [2]. In the past two decades, *Klebsiella pneumoniae* has been identified as the predominant pathogen of PLA in Asia [3]. *Klebsiella pneumoniae*-caused liver abscess (KPLA) was first reported in the 1980s in Taiwan [4], and the incidence of *Klebsiella pneumoniae* causing PLA was from 30% in the 1980s to over 80% in the 1990s among all causal pathogens [5]. KPLA is associated with several risk factors, such as diabetes, biliary tract disease and history of malignancy. Recently, KPLA has been extensively described in mainland China and other countries including America, and Europe [6–8].

*Klebsiella pneumoniae* can colonize the intestine and penetrate the intestinal mucosal barrier in pathological states to enter the liver via the portal vein system and subsequently cause KPLA [3]. KPLA often has a poor prognosis, particularly among those with metastatic infection, including bacteriemia, meningitis, endophthalmitis, and other extrahepatic infections [3, 9]. Research suggests that the rate of metastatic infection has a range of 3.5–20%, and the mortality rate of patients with KPLA is 2.8–10.8% [10]. Sepsis is a life-threatening organ dysfunction caused by a dysregulated host response to an infection, with unacceptably high morbidity and mortality [11]. Sepsis is a common and serious complication in patient with PLA [12]. The delayed diagnose and treatment of PLApatients with initially stable hemodynamics can result in rapid progression to sepsis. Sepsis can lead to organ dysfunction and eventually death.

Unfortunately, little is known about clinical features and the incidence of *K. pneumoniae* causing PLA with sepsis in the Eastern China. Therefore, this study was undertaken to summarize the clinical and microbiologic characteristics, metastatic infection of KPLA patients with and without sepsis.

## Methods

### Study design

This study was a retrospective review of all patients who were diagnosed as KPLA (International Classification of Disease, Clinical Modification 572.0) and hospitalized in the Taizhou Hospital of Zhejiang Province, a 1500-bed tertiary teaching hospital located in east China, from January 2013 to December 2019. The KPLA diagnosis was based on the typical clinical symptoms, such as fever, chills, right upper abdominal pain, combined with imaging examinations of the abscess cavity in the liver, including abdominal ultrasonography (US) and/or computerized tomography (CT) and culture identified with *K. pneumoniae* isolated from the blood or pus samples. The patients with suspected symptoms of sepsis at admission were recruited, and confirmed with blood culture and clinical symptoms. The patient inclusion criteria were as follows: (1) age ≥ 18 years, (2) PLA was the primary cause of the hospitalization but not a complication, (3) diagnosed with KPLA. In this study, we excluded the following patients: (1) the patients without clear records or completed treatment, (2) patients with an amebic liver abscess or fungal liver abscess or parasitic liver abscess. Sepsis was defined according to the Sepsis-3.0 criteria [13]: (1) existing evidence of suspected or confirmed infection; (2) the Sequential Organ Failure Assessment (SOFA) score more than or equal to 2 from baseline or greater than 2 in patients with no baseline score available. Metastatic infection was defined as infection associated with liver abscess developed extrahepatic complication such as endophthalmitis, central nervous system infections, lung abscesses, skin or soft tissue infections, etc. This study protocol was approved by the Institutional Medical Ethics Committee of Taizhou Hospital of Zhejiang Province. Due to the retrospective nature of the study, the need for informed consent was waived.

### Microbiologic data

Pus and/or blood were collected for culture. The VITEK 2 compact system (bioMeìrieux Vitek Inc., France) was used for the identification of the 135 *K. pneumoniae* strains. The antimicrobial resistance of strains was detected by antimicrobial sensitivity tests using VITEK 2 Compact and disc diffusion method on the Mueller-Hinton (MH) Agar plates. All laboratory tests were performed by an accredited laboratory according to ISO 15189:2012 standards. The antimicrobial susceptibility was interpreted according to the latest guidelines publishied by Clinical and Laboratory Standards Institute (CLSI, 2020). These antibiotics included amoxicillin/clavulanic acid, ampicillin/sulbactam, cefazolin, ceftazidime, ceftriaxone, ciprofloxacin, levofloxacin, amikacin, aztreonam, gentamicin, imipenem and meropenem.

### Data collection

Data were collected from the medical record database, including demographic data (age and gender), symptoms and underlying diseases, laboratory and microbiological findings, complications, antimicrobial therapy, necessity of interventional treatment (percutaneous drainage, or surgical drainage) and clinical outcomes (i.e., cured or died). The abscess prognosis was determined in accordance with the criteria based on clinical signs and abscess changes, which were certified by the Chinese Academy of Medical Sciences.

### Statistical analysis

Data entry and processing were conducted using SPSS software, version 20.0 (IBM Inc., New York, USA). Descriptive data were presented as mean ± SD, categorical variables were reported as number and percentage. The Student’s *t*-test and the Mann-Whitney *U* test were applied to evaluate continuous variables. We analyzed categorical variables using the chi-square or Fisher’s exact test. All *p*-values of < 0.05 were considered as statistically significant.

## Results

### Demographic data and comorbidities

During the study period, a total of 135 patients diagnosed as *K. pneumoniae* liver abscess were included. Demographic characteristics, symptoms and underlying diseases of patients with sepsis (*n* = 37) and without sepsis (*n* = 98) were summarized in Table 1. Eighty (59.3%) were males and 55 (40.7%) were females. The mean age at diagnosis of the enrolled patients was 60.9 ± 12.7 years (range 23 to 88). The predominant symptoms in these two groups were fever, chills, abdominal pain. There were significant differences in the following variables in the sepsis group compared to the non-sepsis group: frailty (32.4% vs. 12.2%, *p* = 0.006), diarrhea (18.9% vs. 2.0%, *p* = 0.002). However, the sepsis group was less likely to have abdominal pain (10.8% vs. 55.1%, *p* = 0.000). KLPA patients with sepsis had a higher rate of fatty liver (24.3% vs. 7.1%, *p* = 0.006), chronic renal insufficiency (35.1% vs. 1.0%, *p* = 0.000) and hepatic dysfunction (45.9% vs. 4.1%, *p* = 0.000) (Table 1).

**Table 1:** Demographic characteristics, symptoms and underlying diseases of patients with *K. pneumoniae* liver abscess with sepsis or without sepsis

Characteristic	Total *n* = 135	KPLA with sepsis *n* = 37	KPLA without sepsis *n* = 98	*P* value
Age (year)	60.9 ± 12.7	61.1 ± 12.1	60.8 ± 13.0	0.902
Gender				0.227
Male,n (%)	80 (59.3)	25 (67.6)	55 (56.1)	
Female,n (%)	55 (40.7)	12 (32.4)	43 (43.9)	
Symptoms				
Body temperature (≥38.5 °C)	80 (59.3)	21 (56.8)	59 (60.2)	0.716
Fever	123 (91.1)	35 (94.6)	88 (89.8)	0.511^a^
Chills	81 (60.0)	24 (64.9)	57 (58.2)	0.478
Abdominal pain	58 (43.0)	4 (10.8)	54 (55.1)	0.000
Nausea	17 (12.6)	5 (13.5)	12 (12.2)	0.843
Vomiting	16 (11.9)	7 (18.9)	9 (9.2)	0.119
Frailty	24 (17.8)	12 (32.4)	12 (12.2)	0.006
Diarrhea	9 (6.7)	7 (18.9)	2 (2.0)	0.002^a^
Underlying conditions				
Diabetes mellitus	67 (50.0)	20 (54.1)	47 (48.0)	0.528
Hypertension	51 (37.8)	17 (45.9)	34 (34.7)	0.229
Fatty liver	16 (11.9)	9 (24.3)	7 (7.1)	0.006
Cholelithiasis	29 (21.5)	4 (10.8)	25 (25.5)	0.064
Viral hepatitis	32 (23.7)	7 (18.9)	25 (25.5)	0.422
Cardiovascular diseases	25 (18.5)	5 (13.5)	20 (20.4)	0.358
Chronic renal insufficiency	14 (10.4)	13 (35.1)	1 (1.0)	0.000^a^
Hepatic dysfunction	21 (15.6)	17 (45.9)	4 (4.1)	0.000^a^
Abdominal surgery history	9 (6.7)	0	9 (9.2)	0.063^a^

^a^ Calculated with the Fisher’s exact test

### Imaging and laboratory results

Laboratory findings recorded on admission were shown in Table 2. According to these images, there were no obviously differences between the two groups. Most of the lesions (*n* = 90, 66.7%) were located in the right lobe and the abscess size ranged from 5 cm to 10 cm. Statistically significant differences between patients with and without sepsis were observed in WBC (*p* = 0.015), neutrophil count (*p* = 0.006), platelets (*p* = 0.000), hemoglobin (*p* = 0.026), total bilirubin (*p* = 0.000), blood urea nitrogen (*p* = 0.000), and creatinine (*p* = 0.000).

**Table 2:** Imaging findings and laboratory findings of patients with *K. pneumoniae* liver abscess with sepsis or without sepsis

Characteristic	Total *n* = 135	KPLA with sepsis *n* = 37	KPLA without sepsis *n* = 98	*P* value
Abscess location				
Right lobe	90 (66.7)	25 (67.6)	65 (66.3)	0.891
Left lobe	34 (25.2)	9 (24.3)	25 (25.5)	0.887
Both lobes	11 (8.1)	3 (8.1)	8 (8.2)	1.000^a^
Abscess size (cm)				
< 5	26 (19.3)	5 (13.5)	21 (21.4)	0.298
5–10	85 (63.0)	24 (64.9)	61 (62.2)	0.779
> 10	24 (17.8)	8 (21.6)	16 (16.3)	0.473
Laboratory findings				
WBC > 9.5× 10^9^/L	105 (77.8)	34 (91.9)	71 (72.4)	0.015
NE% > 75%	119 (88.1)	37 (100.0)	82 (83.7)	0.006^a^
PLT < 125× 10^9^/L	38 (28.1)	19 (51.4)	19 (19.4)	0.000
Hb < 110 g/L	37 (27.4)	5 (13.5)	32 (32.7)	0.026
CRP > 10 mg/L	127 (94.1)	37 (100.0)	90 (91.8)	0.106^a^
PCT > 0.5 ng/ml	120 (88.9)	35 (94.6)	85 (86.7)	0.237^a^
ALT > 50 U/L	73 (54.1)	23 (62.2)	50 (51.0)	0.247
AST > 40 U/L	79 (58.5)	25 (67.6)	54 (55.1)	0.190
ALP > 125 U/L	100 (74.1)	26 (70.3)	74 (75.5)	0.535
GGT > 60 U/L	105 (77.8)	30 (81.1)	75 (76.5)	0.571
T.Bil > 20.5 μmol/L	44 (32.6)	22 (59.5)	22 (22.7)	0.000
ALB< 40 g/L	129 (95.6)	37 (100.0)	92 (93.9)	0.188^a^
FIB> 4 g/L	128 (94.8)	33 (89.2)	95 (96.9)	0.089^a^
APTT> 42 s	80 (59.3)	20 (54.1)	60 (61.2)	0.449
BUN > 7.2 mmol/L	41 (30.4)	21 (56.8)	19 (19.6)	0.000
Cr > 97 μmol/L	20 (14.8)	12 (32.4)	8 (8.2)	0.000

*WBC* white blood cell count, *NE%* neutrophil percentage, *PLT* platelets, *Hb* hemoglobin, *CRP* C-reactive protein, *PCT* procalcitonin, *ALT* Alanine Transaminase, *AST* Aspartate Transaminase, *ALP* Alkaline Phosphatase, *GGT* Gamma-Glutamyl Transpeptidase, *T.BIL* Total bilirubin, *ALB* Albumin, *FIB* Fibrinogen, *APTT* Activated Partial Thromboplastin Time, *BUN* blood urea nitrogen, *Cr* creatinine

### Complications and treatments

The complications of KPLA patients with and without sepsis were presented in Table 3. More-frequent development of severe complications including septic shock (35.1% vs. 1.0%, *p* = 0.000) and acute respiratory distress syndrome (10.8% vs. 0, *p* = 0.005) were found in the sepsis group. Twelve (8.8%) patients had metastatic infections. The incidence of metastatic infections of lung (24.3% vs. 2.0, *p* = 0.000) and eye (8.1% vs. 0, *p* = 0.019) was significantly higher in the sepsis group. The mean hospital stay was 14.5 ± 9.0 days. Furthermore, KPLA patients with sepsis experienced a longer hospital stay with 20.7 ± 9.5 days and a higher proportion of ICU admission (29.7% vs. 3.1%, *p* = 0.000). The treatment of KPLA patients included antibiotics alone (5.2%), antibiotics plus percutaneous drainage (90.4%), and antibiotics plus surgical drainage (4.4%). However, there was no significant difference among these groups. The most frequently used antibiotics were carbapenems, followed by third generation cephalosporins, fluoroquinolone, and Beta-lactamase inhibitors, or combined with metronidazole. Compared to the non-sepsis group, the sepsis group had a significantly higher frequency of antibiotic therapy with Beta-lactamase inhibitors (48.6% vs. 20.4%, *p* = 0.001) and carbapenems (83.8% vs. 66.3%, *p* = 0.046). However, no significant difference was found in mortality between the two groups.

**Table 3:** Treatment, complications and outcomes of patients with *K. pneumoniae* liver abscess with sepsis or without sepsis

Characteristic	Total *n* = 135	KPLA with sepsis *n* = 37	KPLA without sepsis *n* = 98	*P* value
Complications				
Sepsis shock	8 (5.9)	7 (18.9)	1 (1.0)	0.000^a^
Acute Respiratory Distress Syndrome	4 (3.0)	4 (10.8)	0	0.005^a^
Acute kidney injury	2 (1.5)	2 (5.4)	0	0.074 ^a^
Pleural effusion	11 (8.1)	4 (10.8)	7 (7.1)	0.493 ^a^
Spontaneous bacterial peritonitis	2 (1.5)	2 (5.4)	0	0.074 ^a^
Metastatic infections				
Lung	11 (8.1)	9 (24.3)	2 (2.0)	0.000^a^
Eye	4 (3.0)	4 (10.8)	0	0.005^a^
CNS	1 (0.7)	1 (2.7)	0	0.274^a^
Others	4 (3.4)	3 (8.1)	1 (1.0)	0.063^a^
Hospital length of stay, days	14.5 ± 9.0	20.7 ± 9.5	12.2 ± 7.7	0.000
ICU admission	14 (10.4)	11 (29.7)	3 (3.1)	0.000^a^
Readmission	8 (5.9)	2 (5.4)	6 (6.1)	1.000^a^
Treatment				0.696
Antibiotics alone	7 (5.2)	1 (2.7)	6 (6.1)	
Antibiotics+Percutaneous drainage	122 (90.4)	34 (91.9)	88 (89.8)	
Antibiotics+Surgical drainage	6 (4.4)	2 (5.4)	4 (4.1)	
Antibiotic drugs				
Beta-lactamase inhibitors	38 (28.1)	18 (48.6)	20 (20.4)	0.001
The third generation of cephalosporin	59 (43.7)	18 (48.6)	41 (41.8)	0.477
Fluoroquinolone	51 (37.8)	17 (45.9)	34 (34.7)	0.229
Carbapenems	96 (71.1)	31 (83.8)	65 (66.3)	0.046
Metronidazole	57 (42.2)	16 (43.2)	41 (41.8)	0.883
Clinical outcomes				0.074^a^
Cured	133 (98.5)	35 (94.6)	98 (100)	
Died (Poor prognosis)	2 (1.5)	2 (5.4)	0	

### Antimicrobial susceptibility

Among the 135 patients, only 4 *K. pneumoniae* strains were ESBL-producing. All the strains were intrinsically resistant to ampicillin, and 8 were unsusceptible to amoxicillin/clavulanic acid, cefazolin, 6 to ampicillin/ sulbactam, levofloxacin, 4 to ceftazidime, ciprofloxacin, aztreonam, gentamicin, 2 to imipenem, meropenem (Table 4). All of these strains were susceptible to amikacin.

**Table 4:** Antibiotics resistance among *K. pneumoniae* isolates

No. of isolates	Amoxicillin /clavulanic acid	Ampicillin/ sulbactam	Cefazolin	Ceftazidime	Ceftriaxone	Ciprofloxacin	Levofloxacin	Amikacin	Aztreonam	Imipenem	Meropenem	Gentamicin
2	R	R	R	R	R	R	R	S	R	R	R	R
2	R	R	R	R	R	R	R	S	R	S	S	R
2	R	R	R	S	S	S	R	S	S	S	S	S
2	R	S	R	S	S	S	S	S	S	S	S	S
127	S	S	S	S	S	S	S	S	S	S	S	S

### Metastatic infection

The characteristics of the 12 patients with metastatic infection are shown in Table 5. Among them, 10 had abscesses in the right hepatic lobe, 2 in the left hepatic lobe. The most frequent complications were septic shock and acute respiratory distress syndrome. Metastatic infection involved multiple sites, such as lung, eye, and central nervous system, which was easy to cause serious consequences, especially in patients with endophthalmitis, and more disabled; and those involving central nervous system were more critical. Four KPLA patients had endophthalmitis, and one patient had central nervous system infection. Eventually, two patients died from complications.

**Table 5:** Clinical details of 12 patients with metastatic infections associated with *K. pneumoniae* liver abscess

Patient	Age, years	Sex	infection site	Underlying disease(s)	Severe complication(s)	Location of abscess	WBC (X10^9^/L)	Neutrophil (%)	CRP (mg/L)	PCT (ng/ml)	Antibiotics history	Outcome
1	60	F	lung	HTN, chronic renal insufficiency	MODF,ARDS,AKI Septic shock,	Left side	12.7	88	136	76	IMP+LEV	Died
2	55	M	Lung peritoneum	DM	Septic shock	Right side	12.6	92	316	157.7	MEM + MXF	Survived
3	58	M	Lung eye	None	None	Right side	14.2	81	74.1	0.49	CRO+ LEV	Survived
4	46	M	Lung eye	HTN, DM, liver cirrhosis	Septic shock	Right side	10	87.7	137.8	7.77	IMP, TZP	Survived
5	82	F	Lung pleura	HTN, Cholelithiasis,Cardiovascular diseases	None	Right side, multiple	15.6	88	104	1.55	MXF + MEM	Survived
6	50	F	lung	chronic hepatitis	MODF,ARDS, Septic shock, DIC	Right side	21.5	96.3	176.7	100	CAZ, MEM + LEV	Survived
7	32	M	Lung	HTN, DM,	Septic shock, ARDS	Right side	11.4	89.6	238.6	8.49	IMP, CAZ	Survived
8	83	F	Lung	Cardiovascular diseases	Septic shock	Right side, multiple	10.6	93.3	311.18	73	CRO, SCF	Survived
9	61	M	Lung CNS eye	HTN, DM, Cardiovascular diseases	ARDS	Right side, multiple	23.4	95.5	113.1	24.2	CRO,TZP, MEM	Died
10	48	M	Lung eye	HTN, DM	None	Right side	33.3	89.5	132.5	20.32	TZP+ LEV	Survived
11	53	F	Lung pleura	HTN, DM, anemia	AKI	Right side	9.3	94.2	267.9	23.25	TZP, IMP, CAZ	Survived
12	53	F	pleura	DM, anemia	None	Left side	10.4	82.5	130.8	25.8	TZP	Survived

*HTN* hypertension, *MODF* multiple organ dysfunction syndrome, *ARDS* acute respiratory distress syndrome, *DIC* disseminated intravascular coagulation, *AKI* acute kidney injury, *TZP* Piperacillin/tazobactam, *LEV* Levofloxacin, *IMP* Imipenem, *MEM* Meropenem, *CAZ* Ceftazidime, *CRO* cefatriaxone, *MXF* Moxifloxacin, *SCF* Cefperazone/sulbactam

### Characteristics and outcome of KPLA patients with or without metastatic infections

As shown in Table 6, clinical manifestations, laboratory findings and outcome of KPLA patients with or without metastatic infections were compared. There were no differences between two groups based on these symptoms and underlying conditions. However, patients with metastatic infections were more likely to suffer chronic renal insufficiency (41.7% vs. 7.3%, *p* = 0.000) than these without metastatic infections. There was a statistical difference in platelets (*p* = 0.015), total bilirubin (*p* = 0.049), blood urea nitrogen (*p* = 0.003) and creatinine (*p* = 0.006), which were higher in the metastatic infections group. In regards to mortality, the metastatic infections group was significantly higher than the non-metastatic infections group (*p* = 0.007).

**Table 6:** Baseline characteristics, clinical presentation, and outcome of patients with *K. pneumoniae* liver abscess with or without metastatic infections

Characteristic	Total *n* = 135	KPLA with Metastatic infection *n* = 12	KPLA without Metastatic infection *n* = 123	*P* value
Age (year)	60.9 ± 12.7	58.3 ± 12.2	61.1 ± 12.8	0.467
Gender				0.494
Male,n(%)	80 (59.3)	6 (50.0)	74 (60.2)	
Female,n(%)	55 (40.7)	6 (50.0)	49 (39.8)	
Symptoms				
Body temperature (≥38.5 °C)	80 (59.3)	7 (58.3)	73 (59.3)	0.945
Fever	123 (91.1)	11 (91.7)	112 (91.1)	1.000^a^
Chills	81 (60.0)	8 (66.7)	73 (59.3)	0.621
Abdominal pain	58 (43.0)	6 (50.0)	52 (42.3)	0.606
Nausea	17 (12.6)	2 (16.7)	15 (12.2)	0.648^a^
Vomiting	16 (11.9)	2 (16.7)	14 (11.4)	0.635^a^
Frailty	24 (17.8)	2 (16.7)	22 (17.9)	1.000^a^
Diarrhea	9 (6.7)	0	9 (7.3)	1.000^a^
Underlying conditions				
Diabetes mellitus	67 (50.0)	7 (58.3)	60 (48.8)	0.528
Hypertension	51 (37.8)	6 (50.0)	45 (36.6)	0.360
Fatty liver	16 (11.9)	1 (8.3)	15 (12.2)	1.000^a^
Cholelithiasis	29 (21.5)	1 (8.3)	28 (22.8)	0.461^a^
Viral hepatitis	32 (23.7)	2 (16.7)	30 (24.4)	0.731^a^
Cardiovascular diseases	25 (18.5)	3 (25.0)	22 (17.9)	0.696^a^
Chronic renal insufficiency	14 (10.4)	5 (41.7)	9 (7.3)	0.000
Hepatic dysfunction	21 (15.6)	3 (25.0)	18 (14.6)	0.399^a^
Abdominal surgery history	9 (6.7)	1 (8.3)	8 (6.5)	0.579^a^
Abscess location				
Right lobe	90 (66.7)	10 (83.3)	80 (65.0)	0.199
Left lobe	34 (25.2)	2 (16.7)	32 (26.0)	0.730^a^
Both lobes	11 (8.1)	0	11 (8.9)	0.598^a^
Laboratory findings				
WBC > 9.5× 10^9^/L	105 (77.8)	10 (83.3)	95 (77.2)	0.628
NE% > 75%	119 (88.1)	12 (100.0)	107 (87.0)	0.359^a^
PLT < 125	38 (28.1)	7 (58.3)	31 (25.2)	0.015
Hb < 110 g/L	37 (27.4)	2 (16.7)	35 (28.5)	0.511^a^
CRP > 10 mg/L	127 (94.1)	12 (100.0)	105 (85.4)	0.367^a^
PCT > 0.5 ng/ml	120 (88.9)	12 (100.0)	108 (87.8)	0.360^a^
ALT > 50 U/L	73 (54.1)	5 (41.7)	68 (55.3)	0.366
AST > 40 U/L	79 (58.5)	7 (58.3)	72 (58.5)	0.989
ALP > 125 U/L	100 (74.1)	10 (83.3)	90 (73.2)	0.443
GGT > 60 U/L	105 (77.8)	8 (66.7)	97 (78.9)	0.332
T.Bil > 20.5 μmol/L	44 (32.6)	7 (58.3)	37 (30.3)	0.049
ALB< 40 g/L	129 (95.6)	12 (100.0)	117 (95.1)	1.000^a^
FIB> 4 g/L	128 (94.8)	11 (91.7)	117 (95.1)	0.487^a^
APTT> 42 s	80 (59.3)	6 (50.0)	74 (60.2)	0.494
BUN > 7.2 mmol/L	41 (30.4)	8 (66.7)	32 (26.2)	0.003
Cr > 97 μmol/L	20 (14.8)	5 (41.7)	15 (12.3)	0.006
Treatment				0.075
Antibiotics alone	7 (5.2)	0	7 (5.7)	
Antibiotics+Percutaneous drainage	122 (90.4)	10 (83.3)	112 (91.1)	
Antibiotics+Surgical drainage	6 (4.4)	2 (16.7)	4 (3.3)	
Clinical outcomes				0.007^a^
Cured	133 (98.5)	10 (83.3)	123 (100.0)	
Poor prognosis	2 (1.5)	2 (16.7)	0	

## Discussion

KPLA is a critical infectious disease, which could cause sepsis and/or other severe complications. However, few studies on the complications of KPLA have been published. In this study, KPLA was diagnosed in 135 of the 360 (37.5%) PLA patients, and the incidence of sepsis in KPLA was 27.4% (37 of 135 patients). Pyogenic liver abscess remains a significant infectious issue for old aged people residing in the rural areas of developing countries. The duration of symptoms prior to admission were over 2 weeks or longer in some of the KPLA patients. The delayed diagnosis and treatment, limited healthcare access may be associated with the higher incidence of sepsis. Our study found that the sepsis group was more likely to have the metastatic infections with lung and eye.

A large proportion of patients in our study were males, with the average age of 60.9 ± 12.7 years old, which was similar to the previous studies [2, 14]. Zhang J et al. reported that elderly patients with age over of 65 years were more likely to develop pyogenic liver abscess [15]. Zhu X et al. pointed out that the mean age of PLA patients was 59.6 years old, and the age group with the greatest number of patients was 51 to 60 years old [16]. In present study, the results showed that men were more likely to develop sepsis than women. Hormonally active women are better protected from sepsis than men. Sex hormones play an important role in inflammatory responses [17]. It was notable that fever was present in 91.1% of patients, which was consistent with other studies [18]. Expectedly, 50.0% of KPLA patients were diabetic, which has been reported previous that diabetes mellitus were the most common underlying disease in KPLA patients [19]. The functional abnormality in neutrophil chemotaxis and phagocytosis may contribute to a relatively high incidence of KPLA in diabetes mellitus. Additionally, we found that sepsis patients had higher incidences of frailty, and diarrhea, which were non-specific and might lead to delayed diagnosis of PLA. Furthermore, patients with sepsis had a significantly higher prevalence of underlying diseases with fatty liver, chronic renal insufficiency, and hepatic dysfunction, indicating that PLA patients with sepsis had a greater incidence of metabolic disorders.

Our findings demonstrated that the laboratory features were also non-specific for the diagnosis of KPLA. Most patients had increased levels of white blood cell counts (WBC), neutrophil percentage (NE%), C-reactive protein (CRP), procalcitonin (PCT), and fibrinogen, which were considered to be the markers for infection. Furthermore, blood urea nitrogen and creatinine levels of the sepsis group were higher, which suggested that renal insufficiency in KPLA patients with sepsis was more evident. In addition, the sepsis group had higher prevalence rates of total bilirubin, probably because of the higher prevalence of liver dysfunction, degree of liver damage. Therefore, these laboratory examinations may reduce the misdiagnosis of PLA.

Extrahepatic metastatic infection is one of the fatal complications for KPLA patients [20]. Recently, *K. pneumoniae* liver abscess with septic metastatic lesions has been often reported [21, 22]. Several studies have been reported that metastatic infections are more common in KPLAs than non-KPLAs, and the prevalence rate of metastatic infection was increased in KPLAs, especially in eye and CNS [9, 23]. In this investigation, we found that the incidence of extrahepatic metastatic infection in KPLA was 8.8%, which was consistent with the previous reports [1, 24]. Importantly, we observed that KPLA patients with sepsis may be more likely to have some complications, including acute kidney injury, acute respiratory distress syndrome, and spontaneous bacterial peritonitis. Furthermore, the sepsis group also revealed a higher incidence of metastatic infection. Our results corresponded to the previous investigations, this may be due to the failure of liquefaction owing to the high prevalence of a phagocytosis-resistant, capsular serotype *K. pneumoniae* associated with liver abscess [25]. In our study, 12 patients with KPLA had severe metastatic infectious conditions at admission. The mortality was 1.5% overall, and 2 of them died of overwhelming sepsis and multiple organ failure. Our data showed that three patients had endophthalmitis. Despite aggressive intravenous and intravitreal antibiotic therapy, 2 of them were eventually eviscerated or enucleated. Other associated septic metastatic infections included pulmonary infection in 11 cases, pleural effusion in 4 cases, brain abscess or meningitis in 1 case, and peritonitis in 1 case.

In the present study, we found that most of the *K. pneumoniae* strains isolated were susceptible to most of the antibiotics, but with resistance to ampicillin only. Previous studies also reported that the emergence of carbapenem-resistant *K. pneumoniae* in some strains may lead to final treatment failure [26]. Therefore, the antibiotics are most widely used in current clinical practice. However, the rising trend in resistance have been reported elsewhere in the world. As we known, the capsule of *K. pneumoniae* may play an important role in the resistance of uptake and killing by host phagocytes [27], it is prudent to ensure sensitivity-directed antibiotics therapy during KPLA treatment to prevent further development of antibiotic resistance.

In general, therapeutic strategies were dependent on the size and number of abscesses, degree of abscess liquefaction, and with/without other possible complications. In our study, the first treatment was antibiotics and percutaneous pigtail catheter drainage of KPLA, followed by antibiotic alone. All these patients received intravenous antibiotics and 90.4% of patients underwent percutaneous drainage, 4.4% underwent surgical drainage. There were no significant differences between these two groups of patients in the treatment of PLA. In addition, the most commonly used antibiotics were carbapenems and third generation cephalosporin combined with or without metronidazole. Except for complicated cases, we recognized that appropriate antibiotic coverage and early adequate percutaneous drainage could be considered as the cornerstones in the therapy for KPLA patients. As for KPLA patients with severe infectious symptoms at admission, adequate coverage with empirical antibiotics may be reasonable until culture data are available.

We acknowledged the limitations in our study. Firstly, this was a retrospective, single-center study, and may not be generalizable, as the majority of our patients were Chinese. Secondly, we excluded the patients with liver abscesses, which were demonstrated no growth on either blood or pus cultures. This was probably related to the use of empirical antibiotics treatment prior to blood or pus collection. In this study, these were excluded because it was hard to specify the pathogen. Finally, whether there is any epidemiologic evidence of the relationship between *K. pneumoniae* capsular serotyping or plasmid-associated virulent factor and the clinical manifestations remains to be investigated. However, we believe that these results may be generalized to routine clinical practice in Chinese patients with pyogenic liver abscess.

In conclusion, PLA is a relatively common infectious disease, and the incidence rate of sepsis in KPLA is quite high. KPLA patients with and without sepsis had many distinct clinical features. Metabolic disorders, including fatty liver, chronic renal insufficiency and hepatic dysfunction are common underlying conditions in patients with sepsis. Furthermore, KPLA patients with sepsis had a significantly higher risk of severe metastatic complications, including lung and eye infections. Based on our data, it is necessary to further elucidate the clinical and microbiological features of KPLA, with a focus on septic metastatic infection.

## Data Availability

The datasets analysed during the current study are available from the corresponding author on reasonable request.

## Abbreviations

PLA: Pyogenic liver abscess
KPLA: *Klebsiella pneumoniae*-caused liver abscesses
